# Sensory Processing Differences in Individuals With Autism Spectrum Disorder: A Narrative Review of Underlying Mechanisms and Sensory-Based Interventions

**DOI:** 10.7759/cureus.48020

**Published:** 2023-10-31

**Authors:** Om Patil, Meghali Kaple

**Affiliations:** 1 Medicine, Jawaharlal Nehru Medical College, Datta Meghe Institute of Higher Education and Research, Wardha, IND; 2 Biochemistry, Jawaharlal Nehru Medical College, Datta Meghe Institute of Higher Education and Research, Wardha, IND

**Keywords:** altered neural connectivity, sensory gating, sensory integration therapy (sit), sensory integration, sensory processing, autism spectrum disorder (asd)

## Abstract

Autism spectrum disorder (ASD) is a neurodevelopmental condition characterized by difficulties with social interaction and restricted, repetitive patterns of behavior. Altered sensory processing and perception are considered characteristics of ASD. Sensory processing differences (SPDs) are commonly observed in individuals with ASD, leading to atypical responses to sensory stimuli. SPDs refer to the way in which individuals receive, process, and respond to sensory information from the environment. People with SPDs may be hypersensitive (over-reactive) or hyposensitive (under-reactive) to sensory input, or they may experience fragmented or distorted perceptions. These differences can make it difficult for individuals with SPDs to filter out irrelevant sensory information, and to integrate sensory information from different sources. This study intends to investigate the underlying mechanisms contributing to SPDs in individuals with autism and determine the effectiveness of sensory-based therapies in addressing these challenges. The literature suggests that altered neural pathways, sensory gating dysfunction, and atypical sensory modulation contribute to SPDs in individuals with ASD. Assistive technology, environmental changes, and sensory-based interventions like sensory integration therapy (SIT) have all shown promise in improving sensory functioning and reducing associated behavioral issues. However, further research is needed to improve our understanding of sensory processing in autism and to optimize interventions for individuals with ASD.

## Introduction and background

Autism spectrum disorder (ASD) is a neurodevelopmental disorder that impacts social skills, communication, learning, and behavior. Other neurodevelopmental disorders include Asperger’s syndrome (AS) and pervasive developmental disorder not otherwise specified (PDD-NOS). The new diagnostic criteria for ASD focus on two key domains: social communication impairments and restricted interests/repetitive behaviors. The prevalence of ASD has increased steadily over the past 20 years, from one in 150 children to 1 in 36 children. Hereditary factors, a parent’s history of a psychiatric disorder, pre-term birth, and fetal exposure to psychotropic agents or insecticides are all associated with a higher risk of developing ASD. There are several scales available to assist in the assessment of behaviors and symptoms related to ASD, including the Childhood Autism Rating Scale (CARS), the Autism Spectrum Disorder-Observation for Children (ASD-OC), and the Developmental, Dimensional, and Diagnostic Interview (3di). Approximately 75% of patients with ASD have comorbid psychiatric illnesses or conditions like attention deficit hyperactivity disorder (ADHD), anxiety, bipolar disorder, depression, Tourette’s, and others [1]. Its etiology is complex. In an effort to determine its cause, a number of risk factors, including genetic and environmental variables, have been investigated. Older research has suggested a significant heritability of autism, and hence the majority of studies have concentrated on identifying the underlying genetic causes of autism. These initiatives have improved our understanding of some of the genetic components linked to autism [2]. According to the majority of studies on adult outcomes in ASD, there is very little social integration, little hope for a job, and a lot of mental health issues in this population [3].

People who suffer from autism frequently struggle with emotion dysregulation (ER), which has been linked to a variety of detrimental effects on both mental and physical health. We draw the conclusion that people with ASD experience more ER challenges and consistently self-report or exhibit a less adaptive manner in which they utilize ER strategy based on the examination of prior study findings [4]. Compared to typically developing (TD) children, gender disparities among ASD children in terms of sensory processing are more pronounced, with females exhibiting more severe symptoms in the subscales measuring hearing, balance, and motion. Additionally, a linear discriminant analysis demonstrated that the sensory processing measure (SPM) and SPM-Preschool (SPM-P) are effective at differentiating TD children from children with ASD, with females performing more accurately than men. Overall, these findings imply that the sensory processing challenges in ASD may have gender-specific features [5].

Sensory processing differences (SPDs) linked to ASD have become a focus of specialized evaluation and treatment, as evidenced by their inclusion in the revised Diagnostic and Statistical Manual of Mental Disorders, Fifth Edition (DSM-5) diagnostic criteria. Strong interests and strong aversions may be present in sensory symptoms. Interventions are frequently aimed at aversions or challenges, supplying sensory input within adaptive frameworks, or perceived evaluating limitations with the aim of enhancing people's capacity to interact with their environments. For instance, a child with ASD might find it difficult to tolerate certain stimuli, such as touch, bright lights, food textures or apparel, certain noises (such as a baby wailing), regular activities (like brushing one's teeth or hair), or more eccentric stimuli, such specific colors. Children's abilities to take care of themselves, leave the house, participate in interventions like school, and interact socially can all be greatly hampered by these sensitivities. As part of their play or other activities with a sensory component, children may also exhibit hyperfocus, which is sometimes referred to as "sensory-seeking" or "stimming" behaviors. Occupational therapists, teachers, parents, and other licensed professionals, among others, can apply sensory-focused interventions in a variety of ways. Although their definitions vary, these therapies frequently incorporate sensory experiences (e.g., heavy clothes or materials, interventions that produce aural sensations) to influence a range of outcomes, including language and adaptive behavior [6].

## Review

Methodology

**Figure 1:**
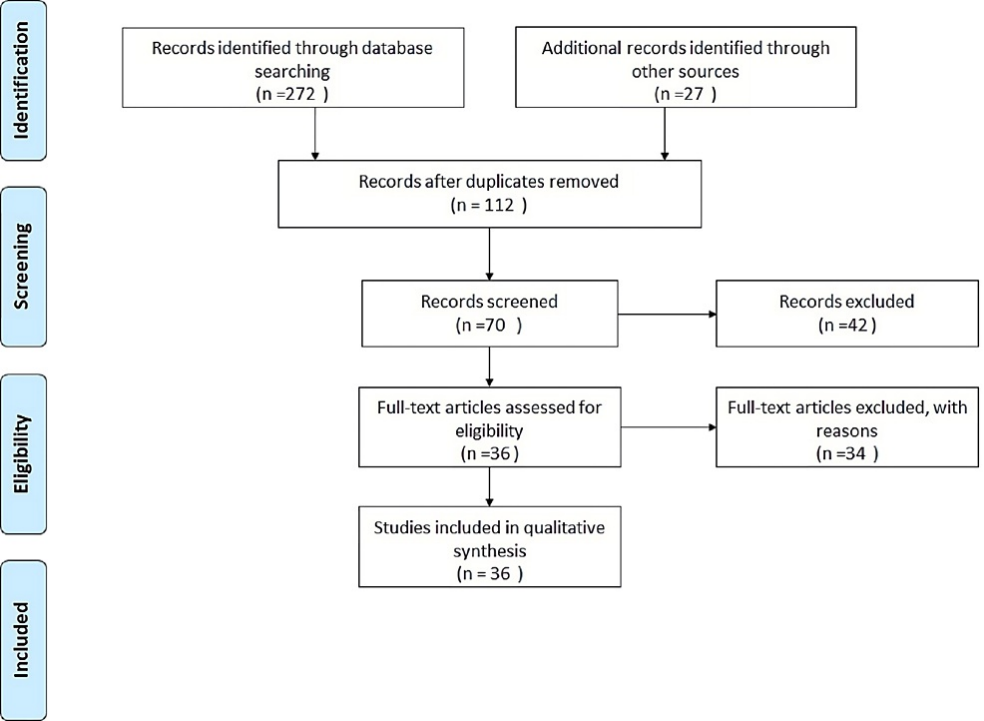
PRISMA flowchart depicting search methodology PRISMA: Preferred Reporting Items for Systematic Reviews and Meta-Analyses

We searched Medline via PubMed and CENTRAL DATABASE via the Cochrane Library for this Narrative Review for the purpose of this review. The search was performed by using the following keywords: altered neural connectivity, sensory gating, sensory integration therapy (sit), sensory integration, sensory processing, and ASD. Furthermore, we screened the reference lists of the potentially relevant studies to seek additional studies. Studies retrieved from these electronic searches and relevant references included in the bibliography of those studies were reviewed. Figure 1 illustrates the selection process of articles used in our study. The records spanning the period 1999-2023 were studied in order to show the journey and advancement of research. We used mainly articles in the English language that were published in international literature, especially in the PubMed library. We aimed to provide data on SPDs, underlying mechanisms, and sensory-based interventions in individuals with ASD.

Underlying mechanisms of sensory processing differences in autism spectrum disorder

Sensory and social processing can take place at many different levels. At a fundamental level, our sensory receptors are triggered by environmental cues. These signals are then transmitted to the brain, where they are processed into a subjective neural image known as perception [7].

Altered Neural Connectivity

Functional findings suggest that ASD patients have an under-connectivity of long-distance connectivity, with a particularly significant deficit of fronto-posterior connections. Structural connectivity indicates disruption of inter-hemispheric white matter structure. Functional studies suggest that ASD patients also have an over-connectivity of local connections, but structural studies are much more heterogeneous. Converging functional connectivity abnormalities in autism with white matter abnormalities suggests that alterations to neural connectivity and communication between brain regions may contribute to behavioral and cognitive dysfunctions associated with autism [8].

In recent studies of autism, functional abnormalities in the default network have been observed during a state of passive rest. Because the default network also plays a role in social processing, emotional processing, and introspection, dysfunction in this network may contribute to some of the challenges that people with autism face in these broad areas [9]. People with ASD have a cognitive advantage when it comes to tasks that involve processing information in detail. This cognitive advantage is thought to be due to atypical neural connections and impaired communication between the two hemispheres [10].

Sensory Gating Dysfunction

Sensory gating is a broad term that refers to the brain’s ability to regulate its response to incoming sensory information. This broad definition makes it possible to define sensory gating as both the ability to reduce or cease response to irrelevant incoming stimuli (gating out) and the ability to respond to novel stimuli (gating in) when a new stimulus is introduced or there is a change in an ongoing stimulus [11]. Auditory P50 sensory gating refers to the activity of the brain to suppress an elicited response to a short auditory stimulus presented immediately after another similar stimulus [12]. Altered sensory gating can lead to sensory overload. N100 suppression deficits in ASD participants were significantly greater than in typically developing control (TDC) participants, as indicated by a larger S2 amplitude, a smaller S2-to-S1 ratio, and a difference between S2 and S1 amplitudes. The amplitude of N100 S2 was significantly related to sensory sensitivity independent of diagnosis. There was no significant group difference in suppression of P50, but S1 amplitude was positively correlated with social deficit in ASD. P200 gating parameters correlated with attention-switching difficulties. These findings suggest that N100 gating deficiency is present in adolescents and young adults with ASD [13]. ASD children have a significantly lower rate of habituation for both audiovisual and visual stimuli compared to neurotypical (NT) children. These results suggest that impaired habituation is a modality-wide issue in ASD. The rates of habituation correlate with several clinical scores related to competence across various phenotypic dimensions, suggesting that sensory difficulties in autism may be related to reduced habituation and associated with clinical symptomology [14].

Atypical Sensory Modulation

Atypical sensory modulation (ASM) is a condition in which the body reacts too much or too little to sensory information in one or several sensory systems. Poor sensory information processing can lead to anxiety [15]. People with autism have atypical reactions to sensory stimulation, which means that they have a different way of processing sensory information. These reactions are different from each other and within the same person with ASD, which can lead to different functional abilities in everyday life [16]. ASD is characterized by abnormal sensory responses, which are listed in the DSM-5. Studies have demonstrated altered sensory processing in individuals with ASD [17].

Some visual disturbances make it hard to read and have been found to be caused by visual stress, also known as ‘pattern-related visual stress’. Reading rate and reading accuracy were measured using the Wilkins Rate of Reading test with and without Intuitive overlays. Children with ASD had a higher incidence of atypical sensory behaviors and visual stress symptoms compared to TDC children [18]. In ASD, there are three main sensory patterns: hypo-response, hyper-response, and sensory seeking [19].

Imbalance in Sensory Excitation and Inhibition

The hypothesis of imbalance between excitation and inhibition (E/I) suggests that there is a disturbance in the levels of both cortical excitation and inhibition in individuals with autism [20]. The imbalance between excitability and inhibition and the increase in E-I (excitatory-inhibitory) ratio is one of the most common mechanisms of learning and memory impairment, cognitive impairment, sensory impairment, motor impairment, and seizures in ASD. E-I imbalance in ASD is mainly caused by a lack of normal neurotransmission in important areas of the brain like the cerebellum, the neocortex, the hippocampus, and the amygdala. Other things that can cause E-I imbalance include a lack of neurotransmitters like oxytocin, a lack of proteins like neuroligins, and a lack of immune system molecules like cytokines. At the neurological level, the E-I imbalance in ASD is called "minicolumnopathy." E-I imbalance changes the way the brain stores information and controls behavior [21]. The pathway known as the corticostriatal pathway serves as a vital conduit for transmitting sensory, motor, and limbic information to the striatum. Its significance lies in its involvement in crucial functions such as motor control, action selection, and reward. Autism-related genes have been linked to abnormal axonal growth, an imbalance in the ratio of excitation to inhibition in the brain, and changes in the long-term plasticity of neurons in the brain's central nervous system [22].

Atypical Multisensory Integration

Atypical multisensory processing (AMP) is one of the candidate basic functions associated with social communication dysfunctions in ASD [23]. The utilization of multisensory processing can bring about a variety of social and cognitive benefits. For instance, the capacity to recognize and respond to stimuli is improved by the utilization of multiple senses [24]. A study focused on the association between multisensory integration and the time-binding window (TBW) of multisensory processing in adults with ASD. Compared to the typically developing group, the ASD group was not as likely to see the illusory flash caused by multisensory integration during the SIFI (sound-induced flash illusion) task. Both groups had similar TBWs in the multisensory temporal order judgment task. Correlation analysis and Bayes factors showed moderate evidence that the decreased SIFI sensitivity was related to the narrow TBW seen in the ASD group. These findings mean that the people with ASD had an atypical way of integrating their sensory information and that different people might have different levels of success in this process depending on how quickly they process their multisensory info [25].

Sensory-based interventions

This type of intervention is based on the idea that sensory processing is a key part of all learning and development and that different ways of processing sensory information in kids with autism can have a ripple effect on how they learn and behave in a variety of areas, including key autism traits [26-28].

Sensory Integration Therapy (SIT)/Ayres Sensory Integration 

Dr. A. Jean Ayres, an occupational therapist, was the first person to conceptualize the theories and therapies associated with sensory integration (SI) as a means of addressing these sensory-based behavior disorders. Her work drew on the neurological knowledge of the 1970s. Since then, the development of neuroimaging technologies has enabled a greater understanding of these areas of the brain [29]. The theory of Ayres Sensory Integration is used to describe behavior, plan interventions, and predict changes in behavior as a result of interventions. They identified the three major parts of sensory integration as describing the typical development of sensory integration, identifying and treating sensory integration dysfunctions, and providing guidance for inter-treatment programs. Understanding these three parts of Ayres Sensory Integration will help occupational therapy professionals to effectively and efficiently implement this approach [30]. SIT is a form of therapy or treatment delivered by qualified occupational therapists who employ play-based sensory-motor activities and the correct challenge to modify the child's sensory responses, thereby reducing distress and enhancing motor function, adaptive responses, focus, and social interaction [31]. One of the most widely implemented interventions in autism is sensory integration; however, there is no consensus among professionals regarding the evidence on which it is based [32].

Environmental Modifications

About 80% of kids with autism are estimated to have sensory processing disorder. Dimming lights, creating a dedicated area for sensory breaks, removing visual distractions, using sensory-friendly kits, helping children with sensory processing problems, placing your child in an optimal space that encourages focus, and helping children with autism to tolerate extra, and minimal distractions are some general modifications that can be made to create a sensory-friendly environment because making these changes are important for the following reasons: it promotes work-life balance, reduces stress for families, encourages meaningful social activities for children and families with autism, enhances work-related performance in activities and tasks, and promotes positive play and social interactions for children and families [33].

Assistive Technologies

One of the major research areas within the field of ASD products is assistive technology development. Mid and advanced products incorporate interactive and intelligent functions with multi-sensory reinforcements to create a more intuitive, adaptive, and dynamic user experience. These products play an extremely important role in enhancing the skills of children with autism, such as working together, developing social skills, and improving their overall well-being [34].

**Table 1:** Summary of underlying mechanisms of SPDs and sensory-based interventions ASD: autism spectrum disorder; SPD: sensory processing difference

Topic	Subtopic	Key points
Underlying mechanisms of SPDs	Altered neural connectivity	· A significant deficit in fronto-posterior connections
		· Neural connectivity alterations contributing to ASD behavioral and cognitive dysfunctions
	Sensory gating dysfunction	· Sensory gating is the regulation of the brain's reaction to incoming sensory stimuli
		· Altered sensory gating can lead to sensory overload
	Atypical sensory modulation	· The body reacts either excessively or inadequately to sensory information
		· Poor sensory information processing can lead to anxiety
Sensory-based interventions	Sensory integration therapy (SIT)	· Describing, identifying, and treating sensory integration dysfunctions
		· SIT is one of the most widely utilized interventions in autism
	Environmental modifications	· Create a sensory-friendly environment for children with autism
		· These changes reduce stress on children and families
	Assistive Technology	· Assistive technology is essential in improving the abilities and skill development of autistic children
		· One of the leading research areas within the field of ASD

Socially assistive robotics (SARs) constitute a promising way to improve the social skills of kids with ASD. In recent years, research has shown that using robots as collaborators can have a positive impact on the social skills development of kids with ASD, particularly in areas where they show significant gaps [35]. XpressiveTalk is a near-imaging avatar that can generate a video of a human face speaking inputted text in a wide range of emotional tones. Results show that general population adults are able to accurately interpret the emotions portrayed by the XpressiveTalk avatar. Adults with autism spectrum conditions have significantly lower accuracy than typical individuals but are still above the chance level for inferring emotions using XpressiveTalk [36].

## Conclusions

People with autism have a wide range of SPDs that can really affect their day-to-day lives and how well they function. These differences can be caused by things like changes in neural connectivity, gating dysfunction, or atypical modulation. Some of the best ways to help with sensory issues in people with autism are through sensory-based therapies, changes in the environment, and help with assistive technologies. But more research needs to be done to figure out what works best, how people with autism differ, and if these interventions will work in the long run. Continued research in this area is of utmost importance because it holds the potential to create improved and individualized interventions that can provide support to individuals with ASD. By delving deeper into the understanding of sensory processing differences and the mechanisms that underlie them, and using evidence-based methods, healthcare professionals can help people with autism have better sensory experiences and we can strive toward a future where individuals with ASD can live in a world that is more inclusive and accommodating to their needs.
